# Bioprocessing of Agricultural Residues as Substrates and Optimal Conditions for Phytase Production of Chestnut Mushroom, *Pholiota adiposa*, in Solid State Fermentation

**DOI:** 10.3390/jof6040384

**Published:** 2020-12-21

**Authors:** Kritsana Jatuwong, Jaturong Kumla, Nakarin Suwannarach, Kenji Matsui, Saisamorn Lumyong

**Affiliations:** 1 Department of Biology, Faculty of Science, Chiang Mai University, Chiang Mai 50200, Thailand; aew.kritsana@gmail.com (K.J.); jaturong_yai@hotmail.com (J.K.); suwan.462@gmail.com (N.S.); 2 Research Center of Microbial Diversity and Sustainable Utilization, Chiang Mai University, Chiang Mai 50200, Thailand; 3 Graduate School of Sciences and Technology for Innovation, Faculty of Agriculture, Yamaguchi University, Yamaguchi 753-8515, Japan; matsui@yamaguchi-u.ac.jp; 4 Academy of Science, The Royal Society of Thailand, Bangkok 10300, Thailand

**Keywords:** fungal phytase, lignocellulosic biomass, response surface methodology, solid state fermentation

## Abstract

Phytase is an enzyme that breaks down phytates to release phosphorus in an available form. This enzyme plays an important role in animals, especially monogastric animals. It serves to improve phytate digestion along with phosphorus absorption, which are required for optimal growth performance and health. In this study, five mushroom species (*Amauroderma rugosum* SDBR-CMU-A83, *Ganoderma mastoporum* SDBR-CMU-NK0244, *Marusmius* sp.1 SDBR-CMU-NK0215, *Pholiota adiposa* SDBR-CMU-R32 and *Piptoporellus triqueter* SDBR-CMU-P234) out of 27 mushroom species displayed positive phytase production by agar plate assay. Consequently, these five mushroom species were selected for determination of their potential ability to produce phytase under solid-state fermentation using five agricultural residues (coffee parchment, oil palm empty fruit bunches, rice bran, sawdust, and water hyacinth) as substrates. The highest yield of phytase production (17.02 ± 0.92 units/gram dry substrate) was obtained after one week of fermentation. Optimization for phytase production was determined by statistical approaches using a Plackett–Burman design to screen ten parameters of relevant substrate components. Two significant parameters, the amount of water hyacinth and the moisture content, were found to affect the production process of phytase. Furthermore, the optimal temperature, pH value, and fermentation period were evaluated. The results indicated that the highest degree of phytase production at 53.66 ± 1.68 units/gram dry substrate (3.15-fold increase) was obtained in water hyacinth containing 85% moisture content by addition with a suitable basal liquid medium at a pH value of 6.5 after being incubated at 30 °C for seven days. The crude phytase of *P. adiposa* was precipitated and the precipitated extract was then used to determine partial characterizations. The precipitated extract displayed high activities after exposure to conditions of 42 °C and pH 5.0. Furthermore, Fe^2+^ enhanced phytase activity and precipitated extract displayed the best stability at a pH value of 8.0 and a temperature of 4 °C.

## 1. Introduction

The agriculture sector of the food production industry has grown significantly over recent decades due to a rapidly growing global population. However, population expansion has also resulted in increases in large amounts of agricultural and agro-industrial residue every year [1]. Between 2003 and 2013, Asia was the largest producer of agricultural residue (47%) followed by America (29%), Europe (16%), Africa (6%), and Oceania (2%) [2]. Agricultural residues are defined as unneeded organic materials that are produced as by-products from harvesting and processing of agricultural crops. Ultimately, agricultural residues are associated with a number of global environmental pollution problems such as those associated with soil and water pollution [3,4]. However, these residues are lignocellulosic in nature and are composed of cellulose, hemicellulose, and lignin waste that include organic acids, salts, and minerals [5,6]. Remarkably, agricultural residues are highly nutritious and can facilitate microbial growth. Therefore, they can potentially be used as raw materials in the production of biofuels, vitamins, antioxidants, animal feed, antibiotics, and other chemicals through solid-state fermentation (SSF) [4]. Furthermore, agricultural residues can be converted into value-added products such as industrially important enzymes that can serve as cheap energy sources for microbial fermentation and enzyme production [7,8,9]. Fungi are a highly potent source for enzyme production due to their excellent capacity for the secretion of extracellular enzymes in breaking down their growth substrate [10,11,12]. Several previous studies involving different agricultural residues (oat husk, rice bran, sawdust, sorghum straw, soybean meal, sugarcane bagasse, and wheat bran) as growth substrates for the production of lignocellulosic enzymes (cellulase, xylanase, laccase), protease, and phytase by fungi under SSF have been conducted [4,12,13,14].

Phytase is an industrially important enzyme that is used in monogastric animal (poultry, swine, and fish) feeds. Phytase (myo-inositol hexaphosphate phosphohydrolases) belongs to the group phosphatase, which catalyzes the hydrolysis of phytate to inositol and free orthophosphate [15]. Phytase is extensively found in various life forms such as plants, the tissue of animals, and especially, microorganisms (bacteria, yeast, and filamentous fungi). The phytase produced by microorganisms has significant commercial potential as it can be used in the animal feed industry [16,17]. Various studies of phytase production from filamentous fungi by SSF using agricultural byproducts as an alternative substrate have been investigated. For example, several filamentous fungi, such as *Aspergillus ficuum*, *A. flavus*, *A. niger*, *A. tubingensis*, *Ganoderma applanatum, G. stipitatum*, *Grifola frondosa*, *Mucor* *racemosus*, *Penicillium purpurogenum*, *Rhizopus* *oligosporus*, *R. oryzae*, *Schizophyllum* *commune*, *Thermomyces lanuginosus*, and *Trametes versicolor,* have been reported as active producers of phytase under SSF when they are fermented using various agricultural residues (wheat bran, wheat straw, soybean meal, rice bran, oil cakes, corn cobs, corn bran, and coconut oil cakes) as substrates [18,19,20,21,22,23,24]. The objectives of our study were to screen for effective phytase-producing mushroom strain under agar plate assay and to select and agricultural residues for phytase production under SSF. The most effective mushrooms strain and the most suitable substrate for phytase production were selected to determine the optimal conditions of the bioprocess of phytase production. This was accomplished by statistical approaches that included Plackett-Burman Design and Central Composite Design. Moreover, the optimal conditions (temperature, pH) and stability (temperature, pH, and metal ions) on phytase activity of precipitated extract were determined. The knowledge gained from this research study can be used to prepare agricultural residues as a growth substrate for phytase production by a selected mushroom strain. This could establish a highly beneficial way to effectively reduce and valorize agricultural residues.

## 2. Materials and Methods

### 2.1. Source of Mushroom Strains

Twenty-seven strains of mushroom mycelia were obtained from the Sustainable Development of Biological Resources Laboratory (SDBR), Department of Biology, Faculty of Science, Chiang Mai University, Chiang Mai, Thailand. The cultures were maintained in 15% glycerol at −20 °C. The isolates were reactivated on potato dextrose agar (PDA) and incubated at 30 °C for 7 days.

### 2.2. Selection of Phytase Producing Mushroom Strains and Phytase Production from Agricultural Residues under SSF

The selection of phytase producing strains was performed using phytase screening agar (PSA), containing (gram per litter) sodium phytate 4, glucose 20, CaCl_2_·2 H_2_O 2, NH_4_NO_3_ 5, KCl 0.5, MgSO_4_·7H_2_O 0.5, FeSO_4_·7H_2_O 0.01, MnSO_4_.7H_2_O 0.01, agar 15; pH 6.0 [18,25]. A mycelial plug (0.5 cm in diameter) obtained from an agar plate of each fungi cultivated on PDA was inoculated onto the modified PSA. The presence of a clear zone around the colony was considered a positive indicator of phytase production. Three replications were generated. Positive phytase producing strains were selected to evaluate the production of phytase under SSF using different agricultural residues as substrates. 

Five agricultural residues, including coffee parchment, oil palm empty fruit bunches, rice bran, sawdust, and water hyacinth, were collected from Chiang Mai, Thailand and used in this study. Five grams of each dried substrate were placed in 125 mL Erlenmeyer flasks. The moisture content of the substrate was adjusted to approximately 80% (w/v) using deionized water. The flasks were then autoclaved at 121 °C for 15 minutes. After allowing the flasks to cool at room temperature for 24 h, five mycelium plugs (0.5 mm diameter) from 5-day-old PDA culture were inoculated and the inoculated flasks were incubated at 30 °C for 7 days. After fermentation, crude phytase was extracted and measured for phytase activity through the process described below. All experiments were performed in triplicate.

### 2.3. Extraction and Determination of Crude Phytase Atcivity 

Crude phytase obtained from fermented agricultural residue with mushroom strains were extracted following the method described by Salmon et al. [18] and Ajith et al. [26] with some modifications. Each fermented agricultural residue was extracted using 25 mL of 0.2 M sodium acetate buffer (pH 5.5) and filtrated through sterilized Whatman’s No.1 paper. The filtrated solution was centrifuged at 6000 rpm at 4 °C for 20 min. The clear supernatant was then collected and used as a crude enzyme. The extraction process was performed under cool conditions (4 °C). 

Phytase activity was determined using the colorimetric assay according to the method described by Harland and Harland [27] with some modifications. In brief, 20 μL of crude enzyme solution was mixed with 110 μL of 6.82 mM sodium phytate in 200 mM sodium acetate buffer (pH 5.5) at 37 °C for 60 min. The enzyme reaction was stopped by adding 500 μL of 10% (w/v) trichloroacetic acid. The development of a blue color was indicative of a positive result for phytase activity. Subsequently, the absorbance was measured at 660 nm and phytase activity was expressed as units per gram dry substrate (U/gds) [28].

### 2.4. Effect of Carbon and Nitrogen Sources for Phytase Production

A suitable mushroom strain (*P. adiposa* SDBR-CMU-R32) and suitable agricultural residue (water hyacinth) were selected in order to optimize the effects of the nutrient sources for phytase production. Fermentation was conducted using the following process. The moisture content of the substrate was adjusted to approximately 80% (w/v) using basal liquid medium (20 g glucose, 2 g CaCl_2_·2H_2_O, 0.5 g KCl, 0.5 g MgSO_4_·7H_2_O, 5 g NH_4_NO_3_, 0.01 g FeSO_4_·7H_2_O, 0.01 g MnSO_4_.7H_2_O, 15 g agar, 1000 mL deionized water, pH 6.0) [18,25]. Deionized water and basal liquid medium in absence of each carbon or nitrogen sources were used as the negative control. Five different carbon sources, including sucrose, fructose, maltose, and corn starch, were used at concentrations of 2.0% by replacing the glucose in the basal liquid medium as has been described above. Moreover, different organic (peptone, tryptone, malt extract, and yeast extract) and inorganic (urea and ammonium sulfate) nitrogen sources at concentrations of 0.5% were used to replace the ammonium nitrate in the basal liquid medium. After seven days of fermentation, the crude enzyme was extracted and phytase activity was measured. Each treatment was performed in three replications. Consequently, suitable carbon and nitrogen sources were selected for further experimentation.

### 2.5. Optimization of Substrate Components and Conditions for Phytase Production Using Statistical Approaches

#### 2.5.1. Optimization of Substrate Component

A Plackett–Burman design (PBD) was used to determine the suitable substrate component for phytase production with a selected mushrooms strain. Glucose (suitable carbon source) and ammonium nitrate (suitable nitrogen source) were used together with other components for the PBD study. A total of ten parameters; glucose (A), ammonium nitrate (B), MgSO_4_·7H_2_O (C), KCl (D), MnSO_4_·H_2_O (E), FeSO_4_·7H_2_O (F), CaCl_2_ (G), water hyacinth (H), moisture content (I), and inoculum (J), were selected and tested as independent variables. The substances were examined at three levels: −1, 0 and +1 for low, medium, and high levels, respectively (Table 1). The maximum levels of A, B, C, D, E, F, G, H, and J were set at 25, 6, 0.1, 0.1, 0.1, 0.1, 2, and 5 g, respectively. The minimum levels of A, B, C, D, E, F, G, H, and J were set at 2, 0.1, 0.1, 0.01, 0.01, 0.01, 0.1, and 0 g, respectively. The maximum and minimum levels of I were set at 5 and 25 mL, respectively. The maximum and minimum levels of J were set at 8 and 12 mycelium plugs, respectively. This experimental design consisted of 20 experiments and 3 central points, totaling 23 experiments. Fermentation was conducted using the process previously described using *P. adiposa*. The enzyme was extracted after 7 days of fermentation at 30 °C and phytase activity was measured. The parameters for which the *p*-value was less than 0.05 were determined to have a significant effect on phytase activity and were chosen for further optimization by respond surface methodology.

#### 2.5.2. Respond Surface Methodology (RSM)

The results from PBD indicated that water hyacinth and moisture content were significant positive factors (*p* < 0.05) on phytase production by *P. adiposa*. In this experiment, the optimum levels were estimated by the CCD of RSM. A CCD consisting of 13 experimental runs with five replications at the center point was employed to determine the effects of the independent variables (Table 2). Fermentation, enzyme extraction and determination of phytase activity were conducted by following the method previously described.

#### 2.5.3. Determination of pH Value of Basal Liquid Medium, Temperature, and Fermentation Period for Phytase Production

A suitable substrate component from previous experiments was used in this experiment for the phytase production of SSF from *P. adiposa*. The pH values of the basal liquid medium were adjusted to 4.0, 5.0, 5.5, 6.0, 6.5, 7.0, 7.5, 8.0, and 9.0 using 0.5 M HCI and 0.25 M NaOH. Each designated pH value was added into a suitable substrate component according to the value of the moisture content in each fermented flask before the flasks were autoclaved. After inoculation, the flasks were incubated at 30 °C for 7 days. The pH value that presented the highest yield of phytase activity was selected for further experimentation. 

The effect of temperature on phytase production was determined. In this experiment, the suitable components and pH values of the basal liquid medium that had been obtained from previous experiments were used. SSF was performed at different temperatures (20, 25, 30, 35, 40, and 45 °C). The crude enzyme was extracted and phytase activity was then measured. The suitable value of SSF for phytase production was selected and used for further experimentation. 

The effect of the fermentation period on phytase production was determined. Suitable substrate component, pH value of the basal liquid medium, and optimum temperature obtained from previous experiments were used. Fermentation was performed for 2 weeks. The crude enzyme was extracted and phytase activity was measured every day. Three replications were prepared for each treatment.

### 2.6. Partial Characterization and Stability of Phytase from Selected Mushroom Strain

#### 2.6.1. Preparation of Precipitated Phytase Extract

Phytase in the crude extract was obtained from SSF of *P. adiposa* under the optimal conditions established in previous experiments. The phytase was then precipitated by saturated ammonium sulfate according to the method described in previous studies [29,30]. The concentration of ammonium sulfate was used in the range of 40%–70%. The precipitation process was performed under cool conditions (4 °C). After 1 h, the precipitate was collected by centrifugation at 6000 rpm at 4 °C for 20 min and stored at –20 °C before being used in further experiments. All treatments in each experiment were carried out in triplicate.

#### 2.6.2. Determination of Optimal Temperature and pH on Phytase Activity

The precipitated extract obtained from a previous experiment was used to determine the optimal temperature for phytase activity. The precipitated extract was dissolved in 0.2 M sodium acetate buffer (pH 5.5) [27]. The enzyme reaction consisted of 20 μL precipitated extract solution and 110 μL of 6.82 mM sodium phytate in 0.2 M sodium acetate buffer (pH 5.5). The enzyme reaction was incubated at different temperatures ranging from 30 to 80 °C. After 1 h of incubation, phytase activity was measured following the method described above. Each treatment was tested in triplicate. In this experiment, one phytase unit was defined as the amount of enzyme to catalyze the release of 1 µmol of inorganic phosphorus per minute under the defined reaction conditions.

The amount of phytase activity recorded at different pH values was determined. Furthermore, 20 μL of the precipitated extract was mixed with 110 μL of 6.82 mM sodium phytate in 0.2 M of different buffering solutions; KCl/HCl buffer for pH 2.0, sodium acetate buffer for pH 3.0–5.5, citrate-phosphate buffer for pH 6.0–7.0, and Tris/HCl buffer for pH 8.0–9.0. The enzyme reaction mixture was incubated at the optimal temperature that had been established in a previous experiment. 

#### 2.6.3. Effect of Cations and Potential Inhibitors on Phytase Activity

The effect of different cations (K^+^, Ca^2+^, Mg^2+^, Mn^2+^, Zn^2+^, Cu^2+^, Fe^2+^, Co^2+^, Na^+^) and potential inhibitors (EDTA, tartrate, citrate, and molybdate) on phytase activity at concentrations of 1 and 5 mM were investigated following the method of Salmon et al. [18]. The activity assayed in the absence of cations and inhibitors was defined as the control. The phytase activity was calculated and expressed as a percent degree of relative activity [31].

#### 2.6.4. Determination of pH and Temperature Stability

The effect of pH on enzyme stability was determined following the modified method of Karthik et al. [23] and Ajith et al. [26]. The precipitate extract was mixed with different buffering solutions; KCl/HCl buffer for pH 2.0, sodium acetate buffer for pH 3.0–5.5, citrate-phosphate buffer for pH 6.0–7.0, Tris/HCl buffer for pH 8.0–9.0, NaHCO_3_/NaOH buffer for pH 10–11, and KCl/NaOH for pH 12 at ratio 1:1 (v/v). The mixtures were incubated at 4 °C. The relative enzyme activity was determined after 12 h and 24 h of incubation.

The effect of temperature on enzyme stability was determined following the method described by Salmon et al. [18] and Spier et al. [19]. The enzyme solution was then stored at different conditions; room temperature (26 ± 2 °C), cooling temperature (4 °C), and freezing temperature (−20 °C). The phytase activity was measured every 2 days until 50 days. The percentage of relative enzyme activity was then calculated. 

### 2.7. Statistical Analysis

Data were analyzed by one-way analysis of variance (ANOVA) using the SPSS version 17.0 for Windows (Chicago, IL, USA). Duncan’s multiple range test was used to determine significant differences between treatments with a *p*–value of less than 0.05. The Design Expert Software version 7.0.0 (Minnesota, USA) was used for statistical analysis of PDB and CCD. The resulting values were then used to design the experiments. The regression and graphical analyses of the experimental data were subsequently obtained.

## 3. Results

### 3.1. Selection of Phytase Producing Mushroom Strains and Agricultural Residue for Phytase Production under SSF

**Figure 1:**
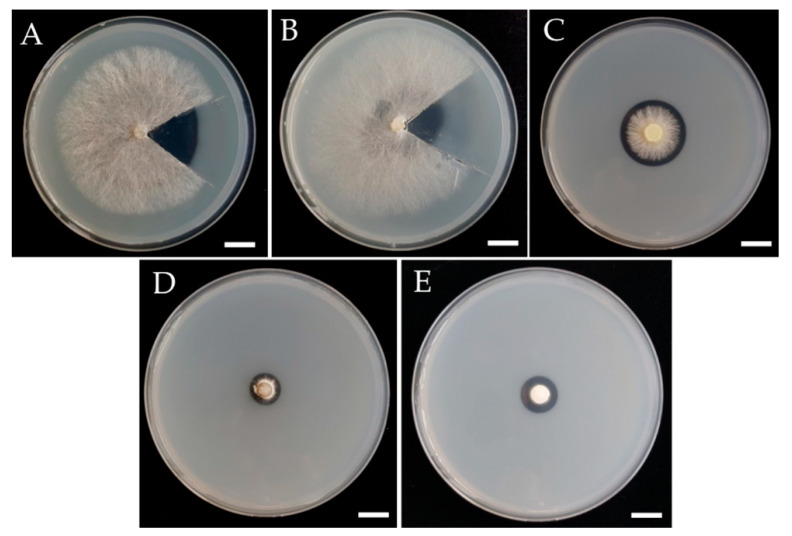
Clear zone of phytase production on PSA. (**A**) *P. adiposa*, (**B**) *G. mastoporum*, (**C**) *Pi. triqueter*, (**D**) *Am. rugosum*, (**E**) *Marasmius* sp.1 Scale bar: 10 mm.

Phytase production was detected on PSA containing sodium phytate as sole source of phosphorus. Among the twenty-seven mushroom strains, only five strains, namely *Am. rugosum*, *G. mastoporum*, *Marasmius* sp.1, *P. adiposa* (chestnut mushroom), and *Pi. triqueter*, were positive for phytase production by the formation of a clear zone (hydrolysis zone) around the colonies (Table 3, Figure 1). Thus, these five mushroom strains were selected for the investigation of phytase production under SSF using different agricultural residues.

**Figure 2:**
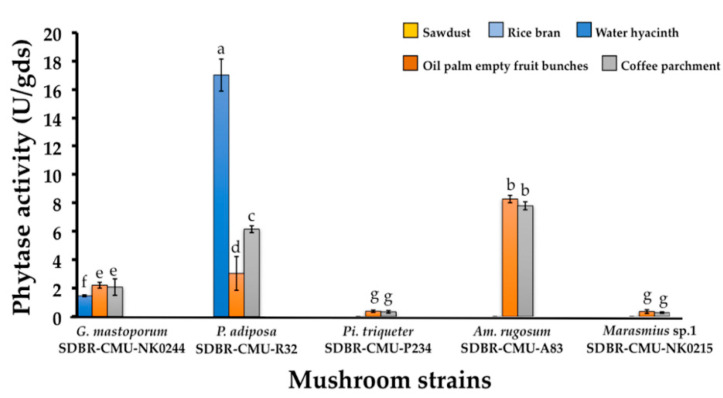
Phytase production during SSF using different agricultural residues by five selected mushroom strains. Five different substrates were tested but only three substrates were found to display phytase activity. The results are means ± SD. Different letters above each bar in the same parameter indicate the significant difference (*p* < 0.05).

Five selected mushroom strains were fermented with five different agricultural residues for phytase production under SSF. It was found that the phytase production of each selected mushroom strain was significantly affected by the agricultural residue that was used. The highest phytase activity of 17.02 ± 1.13 U/gds was achieved with water hyacinth fermented with *P. adiposa*, followed by oil palm empty fruit bunch fermented with *Am. rugosum* (8.28 ± 0.26 U/gds), and coffee parchment fermented with *Am. rugosum* (7.81 ± 0.28 U/gds) (Figure 2). Notably, the lowest phytase activity was obtained from coffee parchment fermented with *Marasmius* sp.1 (0.28 ± 0.05 U/gds). On the basis of the highest phytase activity, *P. adiposa* was selected for further investigation.

### 3.2. Effect of Carbon and Nitrogen Sources in Basal Liquid Medium for Phytase Production

**Figure 3:**
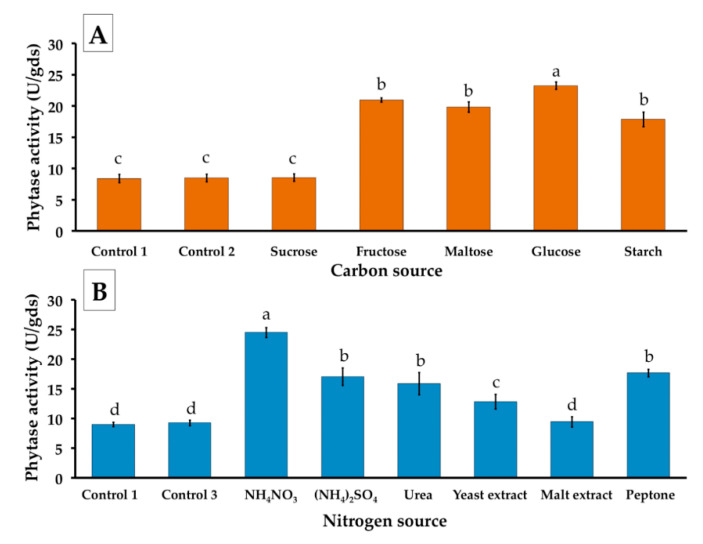
Effect of different nutrient sources for phytase production from *P. adiposa* using water hyacinth in SSF. (**A**) Carbon sources and (**B**) Nitrogen sources. The results are means ± SD. Different letters above each bar in the same parameter indicate the significant difference (*p* < 0.05).

This experiment was performed to examine suitable carbon and nitrogen sources of the basal liquid medium for phytase production from *P. adiposa* under SSF. Water hyacinth was used as a substrate based on the suitable substrate for its phytase production. The results revealed that the highest degree of phytase activity was found in glucose at an average of 23.24 ± 0.37 U/gds, followed by fructose (22.68 ± 0.66 U/gds), maltose (20.07 ± 2.15 U/gds), and starch (19.83 ± 1.17 U/gds) (Figure 3A). The basal liquid medium containing glucose was replaced with different organic and inorganic nitrogen sources. The highest phytase activity was obtained from ammonium nitrate at 24.48 ± 0.3 U/gds, followed by peptone (17.66 ± 0.63 U/gds), and ammonium sulfate (17.04 ± 1.48 U/gds), whereas the lowest phytase activity was found in malt extract (9.42 ± 0.87 U/gds) (Figure 3B). Therefore, glucose and ammonium nitrate were selected and used in the basal liquid medium for further experimentation.

### 3.3. Optimization of Substrate Components and Conditions for Phytase Production by P. Adiposa Using Statistical Approaches

#### 3.3.1. Optimization of Substrate Components

In this experiment, the PBD experimentation included 10 parameters, while 3 levels with a total of 23 experiments were used to select and investigate the optimal level of the variables that influenced the substrate components in phytase production by *P. adiposa*. The values of Prob > *F* of less than 0.05 indicated that the model terms were significant. In this study, water hyacinth (*p* = 0.001) and moisture (*p* = 0.003) were found to be significant variables that influenced the substrate component for phytase production, whereas other variables indicated no significant effects (Table 4). Therefore, the non-influential factors involved in the substrate component on phytase production were used at each minimum level. For future experiments, water hyacinth and moisture were used to find the optimal amounts for the highest level of phytase production using the response surface methodology.

#### 3.3.2. Respond Surface Methodology (RSM)

**Figure 4:**
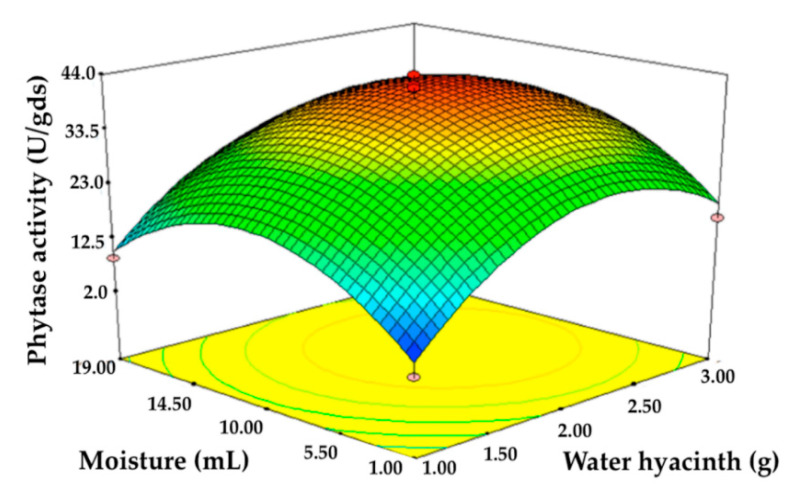
Contour surface plot showing the optimum region and interactions between water hyacinth and moisture content for the phytase production from *P. adiposa*.

The RSM experiment was designed to find the optimal levels of water hyacinth and moisture to achieve the highest yield of phytase production obtained from *P. adiposa*. The final regression equation of the model employing two variables for the evaluation of the highest phytase activity is given in terms of the actual factors in the following model:

$$\mathrm{Phytase}\;\mathrm{activity}\;\left(U/\mathrm{gds}\right)\;=\;-43.14\;+\;58.26A\;+\;3.58B\;-\;0.05\left(A\right)\left(B\right)\;-\;12.81A^{2}\;-\;0.16B^{2}$$

where A is the quantity of water hyacinth (g) and B is the quantity of moisture content (%). After analysis of variance, the quadratic model for phytase activity was found to be significant at *p* < 0.0001 with a reliability rating of R^2^ = 0.9711, while the lack of fit was found not to be significant (Table 5). The interactive effect of water hyacinth and moisture content in the 3D plot showed the optimum level and the maximum yield of the enzyme (Figure 4). According to RSM, the maximum predicted amount of phytase was 40.45 U/gds using 2 g of a suitable substrate component and 85% moisture content, and the produced amount of phytase under this condition was 40.45 ± 2.52 U/gds. The activity was increased by approximately 2.36-fold. Thus, this condition was used for further experimentation.

### 3.4. Determination of pH Value of Basal Liquid Medium, Temperature, and Fermentation Period for Phytase Production of P. adiposa

**Figure 5:**
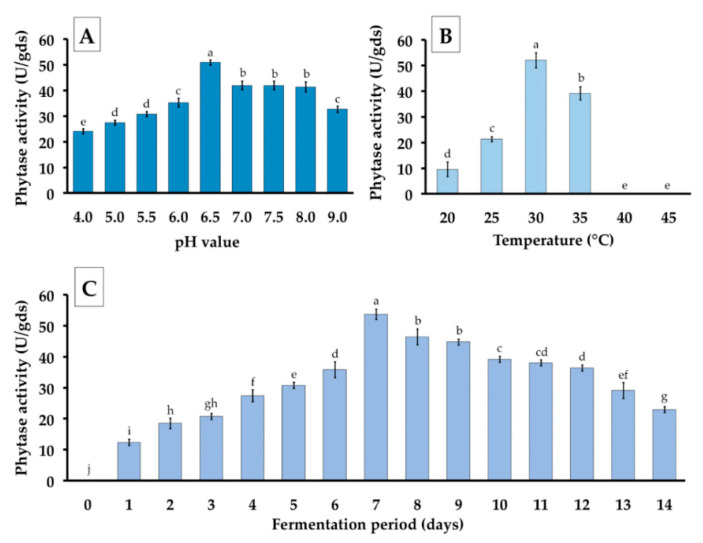
Phytase activity from *P. adiposa* in SSF under different (**A**) initial pH of basal liquid medium, (**B**) temperature for incubation, and (**C**) fermentation periods. The results are means ± SD. Different letters above each bar in the same parameter indicate the significant difference (*p* < 0.05).

The phytase production obtained from *P. adiposa* fermented with a suitable substrate component was investigated under different conditions including an initial pH value of basal liquid medium, selected temperature for incubation, and the appropriate fermentation period. The effect of pH variation of the basal liquid medium for phytase production is shown in Figure 5A. It was found that *P. adiposa* had the ability to produce phytase at pH values ranging from 4.0 to 9.0. A pH of 6.5 was determined to be the optimal pH value of the basal liquid medium at which this mushroom strain produced the highest amount of phytase.

The results indicated that *P. adiposa* grew at tested temperatures ranging from 20–35 °C. The statistical analysis of the data revealed that 30 °C was the best temperature for phytase production and that the highest phytase activity was obtained at 51.98 ± 2.90 U/gds (Figure 5B). However, phytase activity was not observed at 40 and 45 °C due to the fact that this mushroom strain did not grow under that condition.

The effect of fermentation period on phytase production was determined for this mushroom strain. The results showed that the highest amount of phytase activity (53.66 ± 1.68 U/gds) was observed 7 days after incubation at 30 °C and it then slowly decreased (Figure 5C).

Therefore, the optimal condition for phytase production of *P. adiposa* was observed in the suitable substrate components (water hyacinth and nutrients) supplemented with basal liquid medium (containing glucose as a carbon source and ammonium nitrate as a nitrogen source) pH 6.5, containing 85% moisture content, and at 30 °C for seven days. It was found that the selected statistical approach increased phytase activity to 3.15-fold.

### 3.5. Partial Characterization and Stability of Phytase Obtained from P. adiposa

#### 3.5.1. Determination of Optimal Temperature and pH on Phytase Activity

**Figure 6:**
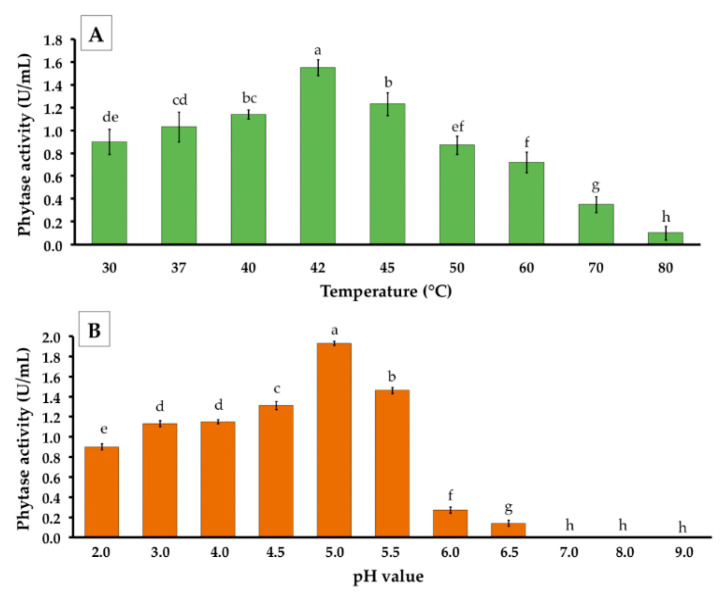
Partial characterization of phytase activity from *P. adiposa* during enzymatic reactions on phytase activity. (**A**) Effect of incubation temperature. (**B**) Effect of different pH values. The results are means ± SD. Different letters above each bar in the same parameter indicate the significant difference (*p* < 0.05).

The obtained precipitate extract after precipitation of the crude enzyme by ammonium sulfate was used for the characterization of phytase activity and stability. It was found that temperatures significantly affected phytase activity. The results indicated that the highest phytase activity (1.55 ± 0.07 U/mL) in the precipitated extract was found at 42 °C (Figure 6A). The activity gradually decreased at a temperature higher than 42 °C. The effect of pH on phytase activity was determined at pH values ranging from 2.0–9.0. The results indicated that pH value affected phytase activity. The highest significant phytase activity (1.93 ± 0.02 U/mL) was found at a pH value of 5.0 (Figure 6B). Notably, phytase activity was not observed at pH values of 7.0, 8.0, and 9.0.

#### 3.5.2. Effect of Cations and Potential Inhibitors on Phytase Activity

The relative activity of the precipitate extract in different cations and inhibitors is presented in Table 6. It was found that Cu^2+^, Zn^2+^, phosphate, and molybdate strongly decreased phytase activity (relative activity value of less than 50%) at concentrations of both 1 and 5 mM. In addition, Mn^2+^, K^+^, Ca^2+^, Na^2+^, Co^2+^, Na^+^, EDTA, tartrate, and citrate exhibited an inhibition effect on phytase activity. However, stimulation of the relative activity of the enzyme was found at concentrations of both 1 and 5 mM of Fe^2+^.

### 3.6. Determination of pH and Temperature Stability

**Figure 7:**
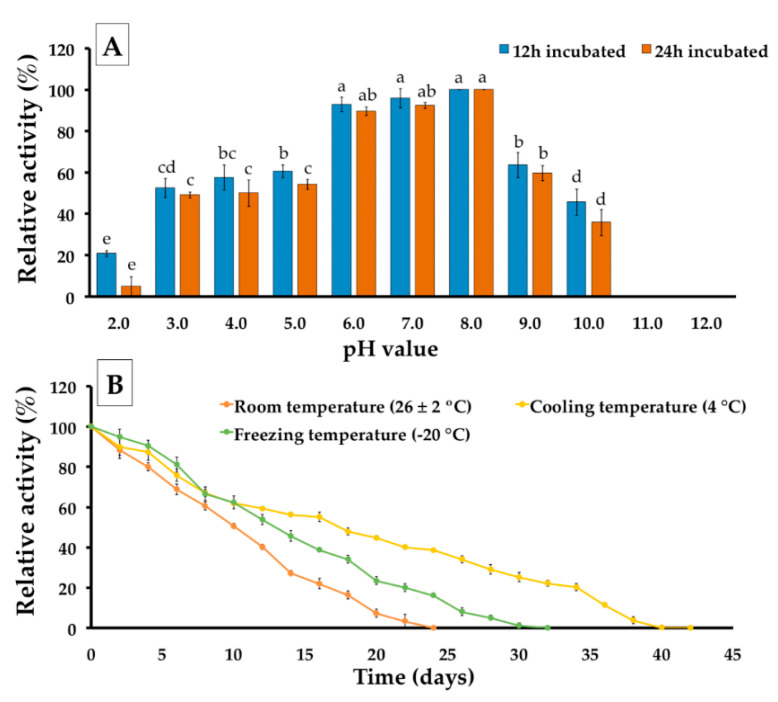
The stability of *P. adiposa* activity in different conditions. (**A**) pH stability during incubation for 12 and 24 h at 4 °C. (**B**) thermal stability. The results are means ± SD. Different letters above each bar in the same parameter indicate the significant difference (*p* < 0.05).

The effect of pH stability on the precipitate extract of *P. adiposa* phytase was determined in pH values ranging from 2.0–12 at 4 °C for 12 and 24 h. The relative activities of the precipitate extract at different pH values are shown in Figure 7A. It was found that the pH value affected phytase activity. The results indicate that the precipitate extract displayed the highest stability at a pH value of 8.0 followed by pH values of 7.0, and 6.0, respectively. The pH values of 2.0 and 9.0 showed a strong decrease in enzyme activity. Notably, the pH values of 11.0 and 12.0 were not observed to have influenced phytase activity.

The temperature stability of the phytase enzyme in the precipitate extract was performed at three different temperatures, namely room temperature (26 ± 2 °C), cooling temperature (4 °C), and freezing temperature (−20 °C), during 50 days of incubation. The results indicated that the relative activity decreased when the length of the incubation period increased (Figure 7B). The degree of relative activity of less than 50% at room temperature, cool temperature, and freezing temperature was observed after incubation for 12, 18, and 14 days, respectively. The results revealed that enzyme activity was lost after incubation at room temperature, freezing temperature, and cool temperature at 24, 32, and 42 days, respectively.

## 4. Discussion

The bioprocess for phytase production by fungi, especially within the genera *Aspergillus*, *Mucor*, *Penicillium*, *Rhizopus*, and *Thermomyces*, using different agricultural residues had been previously studied and reported on by a number of researchers [20,21,23,24,32,33,34,35,36,37]. Nevertheless, only a limited number of published reports are available with regard to the production of phytase obtained from mushroom cultures using agricultural residue through SSF. This present study found that five selected mushrooms strains, namely *Am. rugosum*, *G. mastoporum*, *Marasmius* sp1., *P. adiposa*, and *Pi. triqueter*, effectively produced phytase in cultures on PSA and agricultural residues under SSF. Specifically, the amount of phytase production that was obtained ranged from 1.47 to 17.02 U/gds. Our results indicate that the chestnut mushroom, *P. adiposa*, possessed the greatest phytase producing strains, while water hyacinth was found to be the most suitable substrate for phytase production. These results are similar to those of previous studies [18,38,39] which reported that pure cultures of mushrooms could produce phytase after being cultured with agricultural residues under SSF. Notably, the efficiency of phytase production varied among different mushroom species and strains and was dependent upon the type of agricultural residues being used [18,19,38,39,40,41]. A study conducted by Spier et al. [19] found that the phytase production of *S. commune* DRL−01, *T. versicolor* DRL−18, *G. applanatum* DRL−56, and *G. stipitatum* DRL−70 on wheat bran under SSF ranged from 9.15 to 192.42 U/gds. Salmon et al. [18] found that *S. commune* LPB 101 produced phytase at 113.7 U/gds by SSF using wheat bran. Our findings represent the first report on phytase production by *Am. rugosum*, *G. mastoporum*, *P. adiposa*, and *Pi. triqueter*. Moreover, certain previous studies found that some filamentous fungi in the genera *Aspergillus*, *Mucor*, *Neurospora*, *Pennicillium*, *Humicola*, and *Rhizopus* produced phytase in a range from 16 to 200 U/gds using different agricultural residues under SSF [19,20,21,23,24,42].

The supplementation of different carbon and nitrogen sources had a significant effect on the phytase production of *P. adiposa* under SSF. Our results revealed that glucose and ammonium nitrate can improve the phytase production of *P. adiposa* using water hyacinth. This result was supported by the findings of several previous studies which found that the phytase production of fungi can be influenced by the supplementation of various carbon and nitrogen sources. Our results are similar to those of previous studies [43,44,45,46] which found that glucose and ammonium nitrate supported phytase production under SSF. However, the suitable carbon and nitrogen sources for optimum phytase production were dependent upon both fungal species and strain. Awad et al. [37] found that the highest phytase productivity of *Pen. purpurogenum* GE1 on mixed corn cob and corn bran as substrates was obtained when the specimen was supplemented with glucose and peptone as carbon and nitrogen sources, respectively. Salmon et al. [18] and Gunashree and Venkateswaran [47] studied the effects of different carbon sources for phytase production by *S. commune* LPB 101 and *A. niger* CFR 335 on wheat bran and found that the maximal phytase activity was obtained when using sucrose as a carbon source and yeast extract as a nitrogen source. Furthermore, glucose and ammonium sulfate were indicated as suitable carbon and nitrogen sources for the phytase production of *A. ficuum* PTCC 5288 [24], *A. tubingensis* SKA [43], and *Sporotrichum thermophile* TLR50 [48] through SSF using wheat straw, wheat bran, and sesame oil cakes as substrates, respectively. While Bala et al. [42] found that SSF using wheat bran and sucrose as carbon sources, and ammonium sulfate as a nitrogen source, gave the highest yield of phytase production from *H. nigrescens* BJ82.

A number of factors such as the agricultural residue, nutrient content, the amount of inoculum, and the moisture content can significantly influence the substrate components in the production of fungal enzymes during fermentation [49,50,51,52]. Therefore, the application of various statistical experimental methods by PBD has been used to determine an optimal substrate in order to increase the efficiency of the production of various enzymes by fungi in several previous studies [53,54,55]. According to the statistical results of PDB, the substrate (water hyacinth) and moisture content used in this study were determined to be the primary significant factors on the substrate of phytase production by *P. adiposa* through SSF. This result is similar to previous studies that reported that both substrates and moisture were significant influential factors on the substrate for phytase production by *Aspergillus* species after application of PBD [56,57,58]. Notably, other factors, such as KH_2_PO_4_, (NH_4_)_2_SO_4_, MgSO_4_, FeSO_4_, yeast extract, casein, Tween 80, and glucose, were also found to be influential factors on substrate composition for the phytase production of fungi through SSF using agricultural residues [59,60,61,62,63]. Consequently, the optimal conditions for the phytase production of *P. adiposa* were estimated by CCD using response surface methodology (RSM). Subsequently, the phytase enzyme production increased by 2.36-fold. This finding is supported by the findings of previous studies that indicated that RSM could significantly increase the phytase production of several fungi species under SSF using different agricultural residues [34,64]. For example, Salmon et al. [18] found that the optimal conditions obtained from RSM could significantly increase phytase production in *S. commune* LPB101 using wheat bran as a substrate at an increasing value of 2.85-fold. Phytase production by *A. ficuum* NRRL 3135 and *Ganoderma* sp. MR−56 in wheat bran increased by 1.67-fold and 5.8-fold, respectively after using RSM when compared to non-optimal conditions [39,63]. Spier et al. [58] found that RSM could improve the phytase production of *A. niger* FS3 in citric pulp by increasing enzyme production by 4.3-fold. A study conducted by Wang et al. [57] on phytase production from *A. ficuum* NTG−23 in waste vinegar residues on RSM increased the degree of enzyme production by 7.34-fold. Furthermore, an improved degree of phytase production by RSM was also found in *M. racemosus* NRRL 1994 [62] and *Sp. thermophile* BJTLR50 [59] at increasing values of 1.85-fold and 11.6-fold, respectively.

In this study, the optimum pH value of the supplemented basal liquid medium, temperature, and fermentation period for the highest yield of phytase production from *P. adiposa* were obtained after 7 days of fermentation in culture supplemented with basal liquid medium at a pH value of 6.5 and at 30 °C. This result is supported by the findings of several previous studies that found that phytase production yields were greatly influenced by fermentation conditions (pH value of basal liquid medium, temperature, fermentation period, mineral, aeration, and type of fermentation), while the optimum conditions for phytase production were not found to be homologous for fungal species and strain [65]. The optimum conditions for phytase production from *S. commune* LPB 101 were achieved at three days of fermentation using wheat bran basal liquid medium at a pH value of 7.0 after being incubated at 33 °C [18]. Moreira et al. [66] reported on the effective phytase production of *A. japonicus* URM 5633 in wheat bran using basal liquid medium at a pH value of 6.0 after being incubated at 30 °C for three days. Qasim et al. [41] found that the SSF of *A. tubingensis* SKA in wheat bran supplemented with basal liquid medium at a pH value of 5.0 after being incubated at 30 °C for 4 days resulted in the highest yield of phytase production. Additionally, the yield of phytase production by *Ganoderma* sp. MR−56 was found at four days in wheat bran using basal liquid medium at a pH value of 6.0 and an incubation temperature of 30 °C by submerged fermentation (SmF) [39]. Haritha and Sambasivarao [67] found that the maximum yield of phytase production by *R. oligosporus* MTCC556 was obtained in wheat bran supplemented with basal liquid medium at a pH value of 6.0 after incubation at 30 °C for eight days. Additionally, several previous studies have suggested that the optimal substrate component and physical conditions for phytase fermentation could significantly improve the yields of phytase production on an industrial scale [37,41,68].

Ammonium sulfate precipitation is the method that is the most widely used for the partial purification of phytase from microbial sources [26,69]. Temperature is one of the external factors that plays a significant role in microbial phytase activity. In the present study, the optimal phytase activity of the precipitate extract obtained from *P. adiposa* was achieved at 42 °C. Our findings agree with the results of a number of previous studies which found that the optimal temperature for phytase activity from filamentous fungi occurred within temperatures ranging from 33–50 °C depending upon the fungal species as well as the fungal strain [26,45,47,70,71,72]. However, some previous studies have reported that the optimal temperature for the phytase activity of several *Aspergillus* species, *Ceriporia* sp. CBS 100231, *Mucor hiemalis*, *Peniophora lycii* CBS 686.96, and *Penicillium simplicissimum* W46 was 55 °C [35,73,74,75,76]. Studies on phytase activity involving *A. niger* ATCC 9142 [77] and *A. ficuum* NTG−23 [78] indicated that the optimal temperatures were 65 and 67 °C, respectively. Additionally, the phytases of the thermophilic fungi, *Sp. thermophile* and [48] *Rhizopus microsporus* var. *microsporus* [79], and *R. pusillus* [80] were optimally active at temperatures of 60, 65, and 70 °C, respectively. Furthermore, pH value had a significant effect on the phytase activity of *P. adiposa*. Its phytase activity was achieved at pH values ranging from 2.0 to 6.0, of which 5.0 proved to be the optimal pH value. This result was in accordance with the findings of several previous studies that had reported that the optimal pH value for phytase activity for most fungi was in the acidic range (4.0–5.5). Sanni et al. [70] reported that the optimal phytase activity from *A. fumigatus* was obtained at a pH value of 6.0. Nevertheless, pH values of 7.0 (neutral condition) and 9.5 (basic condition) were found to be optimally active for phytase obtained from *A. flavus* ITCC 6720 [71] and *R. microsporus* var. *microsporus* [79], respectively.

Effect of metal ions and inhibitors on fungal phytase activity was investigated [22,35,81]. In the present study, the activity of phytase from *P. adiposa* decreased in the presence of Ca^2+^, Co^2+^, Cu^2+^, K^+^, Mg^2+^, Mn^2+^, Na^+^, and Zn^2+^ at concentrations of 1 and 5 mM and increased in the presence of Fe^2+^. Studies on the effect of metal ions on phytase activity from *Malbranchea sulfurea* [82] and *Thermomyces lanuginosus* IMI 096218 [22] reported that Fe^2+^ at a concentration of 1 mM increased enzyme activity. Previous reports indicated that metal ions and inhibitors affected fungal phytase activity depending upon the specific type and concentration of the metal ion and inhibitor, as well as the type of fungal phytase [35,83,84]. For example, Soni et al. [83] found that the addition of Hg^2+^ and Cu^2+^ to phytase from *A. niger* NCIM 563 decreased the degree of enzyme activity, whereas Mg^2+^ increased the level of enzyme activity. The activity of partially purified phytase from *A. niveus* was reported to have strongly decreased in 1 mM Mg^2+^, Mn^2+^, Hg^2+^, Cd^2+^, Zn^2+^, and Fe^2+^, but the activity was enhanced by the presence of 1 mM Fe^3+^ [82]. Zn^2+^ and Cu^2+^ at concentrations of 2 mM and inhibited phytase activity from *A. japonicas* BCC18313 and *A. niger* BCC18018 [85]. Phytase activity from *A. fumigatus* PPF−6 was decreased by the addition of 1mM Ca^2+^, Co^2+^, Cu^2+^, Mg^2+^, and Na^+^ was strongly decreased by the addition of 1 mM Ag^2+^, Hg^2+^, Zn^2+^, and Mn^2+^ [86]. The phytase activity from *A. niger* 7A−1 was inhibited by Ca^2+^ [35]. Salmon et al. [18] investigated the effect of metal ions on phytase production by *S. commune* and reported that Ca^2+^, Co^2+^, Cu^2+^, Fe^2+^, Fe^3+^, K^+^, Mg^2+^, Mn^2+^, Ni^2+^, and Zn^2+^ at concentrations of 1 mM increased enzyme activity. Our results indicate that EDTA, tartrate, and citrate decreased the activity of phytase from *P. adiposa*, while molybdate significantly inhibited enzyme activity. This result was in accordance with published findings that revealed that the phytase activity from *S. commune* LPB101 and *R. oligosporus* DSMZ 1964 were decreased by the presence of molybdate [18,87]. Notably, a study conducted by Azeke et al. [87] and Vats and Banerjee [73] on phytase activity from *Aspergillus* species found that fluoride strongly inhibited enzyme activity. In contrast, 1 mM EDTA has been reported to have increased the activity of partially purified phytase obtained from several strains of *A. niger* [73,83].

This study found that the degree of pH stability of the precipitated extract obtained from *P. adiposa* was highly stable within a pH range of 6.0–8.0 and at the low temperature of 4 °C. This result was supported by several previous studies that reported the stability of phytase produced from fungi was influenced by certain conditions such pH and temperature and was dependent upon the fungal species [18,66,88]. Salmon et al. [18] reported that phytase produced from *S. commune* was stable at pH values in a range of 5.0 to 6.0 and at 4 °C. A study conducted by Karaman et al. [88] on the pH stability of phytase obtained from *A. niger* found that it was highly stable at a range of pH values of 5.0 to 6.0. The phytase production from *Monascus* sp., *Neurospora crassa* CICIM F00021, and *Thermomyces lanuginosus* IMI 096218 was highly stable at pH values ranging from 5.0 to 7.0, 6.0 to 7.0, 5.0 to 7.0, and 6.0 to 6.5, respectively. Moreira et al. [66] found that phytase produced by *A. japonicus* URM 5633 could be stable in a pH range of 3.0 to 8.0. Notably, studies on the stability of phytase produced by *Aspergillus* sp. L117, *G. applanatum* DRL−56, *G. stipitatum* DRL−70, *S. commune* DRL−01, *S. commune* LPB101, and *T. versicolor* DRL−18 found them to be stable at 4 °C. The stability of fungal phytase toward different processing conditions, pH values, and temperatures make up the major factors affecting the section and application of fungal phytase [89,90].

## 5. Conclusions

Fungi as a source of phytase production can be a candidate for beneficial applications in biotechnological processes that would facilitate the reduction and valorization of agricultural residues. This would be achieved as a result of the eco-friendly conversion of low-value by-products into potentially beneficial resources and value-added products. In this study, five selected mushroom strains, namely *P. adiposa*, *G. mastoporum*, *Pi. triqueter*, *Am. rugosum*, and *Marasmius* sp.1, produced phytase in various agricultural residues under SSF. Our study obtained high yields of phytase production from *P. adiposa* using water hyacinth under SSF. The optimization for its phytase production was determined by various statistical approaches. The highest phytase yield was observed in the suitable substrate component of water hyacinth containing 85% moisture content along with the addition of a suitable basal liquid medium at a pH value of 6.5 after being incubated at 30 °C for seven days. Enzyme extracts revealed the highest activity at 42 °C and a pH of value of 5.0. Moreover, Fe^2+^ enhanced phytase activity and the precipitated extract displayed the stability at a pH value of 8.0 and a temperature of 4 °C. Further studies on phytase obtained from this mushroom are required to fully process and evaluate its purification and kinetic potential. Additionally, phytase production from selected agricultural residues should be conducted on a larger scale to confirm their potential use in various industries like those associated with the production of food additives and animal feeds, as well as for use in the medical field.

## Figures and Tables

**Table 1:** Experimental design and respond in Plackett–Burman study.

Expt. No.	Independent Variables										Response Phytase Activity (U/gds)	
	A	B	C	D	E	F	G	H	I	J	Experimental	Predicted
1	25(+1)	6(+1)	0.01(−1)	0.01(−1)	0.1(+1)	0.1(+1)	2(+1)	5(+1)	5(−1)	12(+1)	0.92	1.38
2	2(−1)	6(+1)	0.1(+1)	0.01(−1)	0.01(−1)	0.1(+1)	2(+1)	5(+1)	25(+1)	8(−1)	17.24	14.42
3	25(+1)	0.1(−1)	0.1(+1)	0.1(+1)	0.01(−1)	0.01(−1)	2(+1)	5(+1)	25(+1)	12(+1)	7.90	8.10
4	25(+1)	6(+1)	0.01(−1)	0.1(+1)	0.1(+1)	0.01(−1)	0.1(−1)	5(+1)	25(+1)	12(+1)	10.06	6.47
5	2(−1)	6(+1)	0.1(+1)	0.01(−1)	0.1(+1)	0.1(+1)	0.1(−1)	0(−1)	25(+1)	12(+1)	0.000	4.79
6	2(−1)	0.1(−1)	0.1(+1)	0.1(+1)	0.01(−1)	0.1(+1)	2(+1)	0(−1)	5(−1)	12(+1)	0.000	−5.60
7	2(−1)	0.1(−1)	0.01(−1)	0.1(+1)	0.1(+1)	0.01(−1)	2(+1)	5(+1)	5(−1)	8(−1)	0.55	3.31
8	2(−1)	0.1(−1)	0.01(−1)	0.01(−1)	0.1(+1)	0.1(+1)	0.1(−1)	5(+1)	25(+1)	8(−1)	22.56	17.62
9	25(+1)	0.1(−1)	0.01(−1)	0.01(−1)	0.01(−1)	0.1(+1)	2(+1)	0(−1)	25(+1)	12(+1)	0.84	3.63
10	2(−1)	6(+1)	0.01(−1)	0.01(−1)	0.01(−1)	0.01(−1)	2(+1)	5(+1)	5(−1)	12(+1)	0.56	1.97
11	25(+1)	0.1(−1)	0.1(+1)	0.01(−1)	0.01(−1)	0.01(−1)	0.1(−1)	5(+1)	25(+1)	8(−1)	17.89	17.98
12	2(−1)	6(+1)	0.01(−1)	0.1(+1)	0.01(−1)	0.01(−1)	0.1(−1)	0(−1)	25(+1)	12(+1)	0.000	−0.17
13	25(+1)	0.1(−1)	0.1(+1)	0.01(−1)	0.1(+1)	0.01(−1)	0.1(−1)	0(−1)	5(−1)	12(+1)	0.000	−1.34
14	25(+1)	6(+1)	0.01(−1)	0.1(+1)	0.01(−1)	0.1(+1)	0.1(−1)	0(−1)	5(−1)	8(−1)	0.000	−3.69
15	25(+1)	6(+1)	0.1(+1)	0.01(−1)	0.1(+1)	0.01(−1)	2(+1)	0(−1)	5(−1)	8(−1)	0.000	−2.85
16	25(+1)	6(+1)	0.1(+1)	0.1(+1)	0.01(−1)	0.1(+1)	0.1(−1)	5(+1)	5(−1)	8(−1)	0.000	5.38
17	2(−1)	6(+1)	0.1(+1)	0.1(+1)	0.1(+1)	0.01(−1)	2(+1)	0(−1)	25(+1)	8(−1)	0.000	1.09
18	2(−1)	0.1(−1)	0.1(+1)	0.1(+1)	0.1(+1)	0.1(+1)	0.1(−1)	5(+1)	5(−1)	12(+1)	3.20	4.26
19	25(+1)	0.1(−1)	0.01(−1)	0.1(+1)	0.1(+1)	0.1(+1)	2(+1)	0(−1)	25(+1)	8(−1)	0.000	2.56
20	2(−1)	0.1(−1)	0.01(−1)	0.01(−1)	0.01(−1)	0.01(−1)	0.1(−1)	0(−1)	5(−1)	8(−1)	0.000	2.42
21	13.5(0)	3.05(0)	0.06(0)	0.06(0)	0.06(0)	0.06(0)	1.05(0)	2.5(0)	15(0)	10(0)	24.95	23.07
22	13.5(0)	3.05(0)	0.06(0)	0.06(0)	0.06(0)	0.06(0)	1.05(0)	2.5(0)	15(0)	10(0)	23.52	23.07
23	13.5(0)	3.05(0)	0.06(0)	0.06(0)	0.06(0)	0.06(0)	1.05(0)	2.5(0)	15(0)	10(0)	20.73	23.07

A total of ten variables was tested. A = glucose, B = ammonium nitrate, C = MgSO_4_·7H_2_O, D = KCl, E = MnSO_4_·H_2_O, F = FeSO_4_·7H_2_O, G = CaCl_2_, H = water hyacinth, I = moisture, and J = amount of inoculum.

**Table 2:** The experimental design and responses for optimization of phytase activity with *P. adiposa* using CCD.

Run	Independent Variables (g/mL)		Phytase Activity (U/gds)	
	Water Hyacinth (g)	Moisture (mL)	Experimental	Predicted
1	1	1	2.98	5.67
2	3	1	16.60	19.58
3	1	19	8.60	9.98
4	3	19	20.40	22.07
5	0.59	10	7.60	5.63
6	3.14	0	26.39	24.01
7	2	22.37	14.53	11.42
8	2	10	17.48	16.23
9	2	10	43.82	40.45
10	2	10	37.92	40.45
11	2	10	41.94	40.45
12	2	10	37.16	40.45
13	2	10	41.40	40.45

**Table 3:** The list of mushroom strains screened for phytase activity in this study.

Mushroom Species	Strains SDBR-CMU	ITS GenBank Number	Phytase Activity
*Amauroderma rugosum*	A83	MW266980	+
*Auricularia cornea*	A86	MW266993	−
*Ceriporia* sp.	4G	MW281788	−
*Coprinopsis* sp.	NK0217	MW267044	−
*Coprinopsis cinerea*	NS48674−1	MW281768	−
*Cryptomarasmius crescentiae*	R1	MW284415	−
*Cryptomarasmius crescentiae*	R11	MW284413	−
*Cryptomarasmius crescentiae*	R12	MW284414	−
*Daldinia* sp.	D276	MW284416	−
*Ganoderma mastoporum*	NK0244	MW266995	+
*Gloeoporus* sp.	R10	MW281791	−
*Hyphodermella corrugata*	R17	MW281767	−
*Hypoxylon haematostroma*	H145	MW281766	−
*Marasmius* sp.1	NK0215	MW267220	+
*Marasmius* sp.2	NK0218	MW267273	−
*Marasmius sutepensis*	NK0240	MW267298	−
*Pholiota adiposa*	R32	MW281763	+
*Piptoporellus* *triqueter*	P234	MW267951	+
*Pleurotus giganteus*	NK0228	MW267794	−
*Pleurotus pulmonarius*	NK0530	MW266959	−
*Pleurotus sirindhorniae*	NK0130	MT349509	−
*Polyporus* sp.	P235	MW267633	−
*Pycnoporus sanguineus*	NK0189	MW267631	−
*Pycnoporus sanguineus*	P14	MW267632	−
*Resinicium* sp.	R27	MW281793	−
*Trametes cubensis*	An-L1−5	MW267646	−
*Xylaria* sp.	NK0216	MW267256	−

“+” = positive phytase production and “−” = negative phytase production.

**Table 4:** Data analysis (*p*-values and coefficients) of ten variables on phytase production of *P. adiposa* using PBD.

Source	Coefficient	Sum of Squares	df	Mean Square	*F* Value	*p*-Value (Prob > *F*)
Model	-	783.66	10	78.37	4.45	0.011
A-Glucose	−0.33	2.11	1	2.11	0.12	0.736
B-NH_4_NO_3_	−1.21	28.18	1	28.18	1.66	0.225
C-MgSO_4_	0.54	5.77	1	5.77	0.33	0.579
D-KCI	−1.91	73.31	1	73.31	4.16	0.066
E-MnSO_4_	−0.36	2.56	1	2.56	0.15	0.710
F-FeSO_4_	0.36	3.03	1	3.03	0.17	0.686
G-CaCl_2_	−1.28	32.99	1	32.99	1.87	0.199
H-Water hyacinth	4.00	320.38	1	320.38	18.18	0.001
I-Moisture	3.56	253.92	1	253.92	14.41	0.003
J-Inoculum	−1.74	60.40	1	60.40	3.43	0.091
Curvature	-	939.69	1	939.69	53.32	˂0.0001
Residual	-	193.86	11	17.62	-	-
Lack of fit	-	184.65	9	20.52	4.45	0.1968
Pure error	-	9.22	2	4.61	-	-
Co total	-	1917.21	22	-	-	-

**Table 5:** ANOVA analysis of water hyacinth and moisture content for phytase production of *P. adiposa* after fitting with response surface quadratic model.

Source	Coefficient	Sum of Squares	df	Mean Square	*F* Value	*p*-Value (Prob > *F*)
Model		2462.80	5	4.92.56	47.05	˂0.0001
A-Water hyacinth	6.50	337.91	1	337.91	32.28	0.0007
B-Moisture	1.70	23.09	1	23.09	2.21	0.1811
AB	−0.45	0.83	1	0.83	0.079	0.7867
A^2^	−12.81	1142.40	1	1142.40	109.12	˂0.0001
B^2^	−13.31	1232.36	1	1232.36	117.71	˂0.0001
Residual		73.29	7	10.47		
Lack of fit		41.54	3	13.85	1.74	0.2960
Pure error		31.75	4	7.94		
Cor total		2536.06	12			

**Table 6:** Effect of cations and inhibitors on *P. adiposa* phytase.

Treatment	Compound	Relative Activity (%)	
		1 mM	5 mM
Control	Deionized water	100	100
Cations	Mn^2+^	94.0 ± 2.3	41.2 ± 1.5
	K^+^	92.6 ± 2.3	40.7 ± 0.9
	Ca^2+^	90.5 ± 2.3	44.7 ± 2.3
	Mg^2+^	97.6 ± 1.5	55.8 ± 2.4
	Fe^2+^	106.7 ± 1.5	112.3 ± 2.3
	Cu^2+^	36.6 ± 3.1	13.2 ± 0.9
	Zn^2+^	18.8 ± 0.9	8.1 ± 0.9
	Co^3+^	92.5 ± 2.3	40.1 ± 3.2
	Na^+^	93.5 ± 2.3	47.7 ± 3.9
Inhibitors	EDTA	87.9 ± 0.9	44.2 ± 1.5
	Tartrate	97.1 ± 2.3	47.2 ± 1.5
	Citrate	94.5 ± 1.5	45.7 ± 1.5
	Molybdate	17.8 ± 2.3	7.1 ± 0.9
